# A Multi-Parametric Wearable System to Monitor Neck Movements and Respiratory Frequency of Computer Workers

**DOI:** 10.3390/s20020536

**Published:** 2020-01-18

**Authors:** Daniela Lo Presti, Arianna Carnevale, Jessica D’Abbraccio, Luca Massari, Carlo Massaroni, Riccardo Sabbadini, Martina Zaltieri, Joshua Di Tocco, Marco Bravi, Sandra Miccinilli, Silvia Sterzi, Umile G. Longo, Vincenzo Denaro, Michele A. Caponero, Domenico Formica, Calogero M. Oddo, Emiliano Schena

**Affiliations:** 1 Unit of Measurement and Biomedical Instrumentation, Università Campus Bio-Medico di Roma, Via Alvaro del Portillo, 00128 Rome, Italy; d.lopresti@unicampus.it (D.L.P.); arianna.carnevale@unicampus.it (A.C.); c.massaroni@unicampus.it (C.M.); riccardo.sabbadini@alcampus.it (R.S.); m.zaltieri@unicampus.it (M.Z.); j.ditocco@unicampus.it (J.D.T.); 2 Department of Orthopaedic and Trauma Surgery, Università Campus Bio-Medico di Roma, Via Alvaro del Portillo, 00128 Rome, Italy; g.longo@unicampus.it (U.G.L.); denaro@unicampus.it (V.D.); calogero.oddo@santannapisa.it (C.M.O.); 3 Neuro-Robotic Touch Laboratory, BioRobotics Institute, Sant’Anna School of Advanced Studies, 56025 Pisa, Italy; jessica.dabbraccio@santannapisa.it (J.D.); luca.massari@santannapisa.it (L.M.); 4 Department of Physical and Rehabilitation Medicine, Università Campus Bio-Medico di Roma, Via Alvaro del Portillo, 00128 Rome, Italy; m.bravi@unicampus.it (M.B.); s.miccinilli@unicampus.it (S.M.); s.sterzi@unicampus.it (S.S.); 5 Photonics Micro-and Nanostructures Laboratory, ENEA Research Center of Frascati, 00044 Rome, Italy; michele.caponero@enea.it; 6 NEXT Lab, Università Campus Bio-Medico di Roma, Via Alvaro del Portillo, 00128 Rome, Italy; d.formica@unicampus.it

**Keywords:** neck movements, breathing activity, respiratory frequency, occupational health and safety interventions, wearable system, fiber Bragg gratings, flexible sensors

## Abstract

Musculoskeletal disorders are the most common form of occupational ill-health. Neck pain is one of the most prevalent musculoskeletal disorders experienced by computer workers. Wrong postural habits and non-compliance of the workstation to ergonomics guidelines are the leading causes of neck pain. These factors may also alter respiratory functions. Health and safety interventions can reduce neck pain and, more generally, the symptoms of musculoskeletal disorders and reduce the consequent economic burden. In this work, a multi-parametric wearable system based on two fiber Bragg grating sensors is proposed for monitoring neck movements and breathing activity of computer workers. The sensing elements were positioned on the neck, in the frontal and sagittal planes, to monitor: (i) flexion-extension and axial rotation repetitions, and (ii) respiratory frequency. In this pilot study, five volunteers were enrolled and performed five repetitions of both flexion-extension and axial rotation, and ten breaths of both quite breathing and tachypnea. Results showed the good performances of the proposed system in monitoring the aforementioned parameters when compared to optical reference systems. The wearable system is able to well-match the trend in time of the neck movements (both flexion-extension and axial rotation) and to estimate mean and breath-by-breath respiratory frequency values with percentage errors ≤6.09% and ≤1.90%, during quiet breathing and tachypnea, respectively.

## 1. Introduction

Neck pain is a musculoskeletal disorder (MSD) highly prevalent in office works [1,2]. Thirty-one percent of computer workers in Europe experience neck pain [3]. It represents a socio-economic burden since it causes periods of absence from work, reduced productivity, and high utilization of health care services [3,4,5]. Proper occupational health and safety interventions (e.g., ergonomics training and disability management programs) can be sufficient to reduce MSDs’ symptoms and to contrast their negative impacts on society and workplaces [6,7].

Awkward postures may be influenced by bad workers’ habits and by the non-compliance of computer workstations to the ergonomics guidelines. Inadequate positions of monitor, mouse, keyboard, heights of seat and desk can force workers to assume prolonged head flexion-extension (FE) and twisted neck (i.e., when the top of the head tilts to one side while the chin to the other side [8]). Inadequate and prolonged sitting postures, and workers’ stressful conditions can cause a greater activation of the neck muscles [7,9,10,11,12,13]. Besides, alteration of cervicothoracic mobility is correlated to respiratory dysfunctions [1,11,14,15,16,17]. Monitoring respiratory frequency (f_R_) during working activities is of great value because it is sensitive to cognitive load, emotional stress, pain, and discomfort. This parameter has been demonstrated to be related to cognitive load, with important implications for workers exposed to highly demanding tasks [18,19,20].

A potential solution can be the use of systems able to collect quantitative information of both neck postures and respiration. In the literature, only a few systems have been proposed for monitoring wrong postures and breathing activity [21,22,23]. Focusing on the computer working scenario, systems embedding sensors directly in contact with the body (i.e., contact-based systems) are recommended. Such systems do not need structured environments and may allow the performance of job-related activities without limitations. Among contact-based systems, wearables have been proven as an effective and comfortable solution to monitor vital signs (e.g., respiratory frequency, heart rate, blood pressure, and temperature) [21,24,25], posture and human body motion [26,27,28]. A recent technological breakthrough in the area of wearables is promoting innovative applications for spine curvature monitoring [29] using inertial sensors [30,31], strain gauge [32], and optical sensors [33,34,35]. However, despite the great importance of neck posture monitoring to contrast adverse effects caused by long-term wrong postural habits (e.g., reduced productivity, absence from work and high utilization of health care services) only a few studies presented wearable sensors to monitor cervical spine movements [1,36,37]. Recent works proposed wearables based on piezoresistive sensors [38] and fiber Bragg grating (FBG) sensors [39] for monitoring single plane neck movements.

In this work, we developed the first wearable system able to monitor both neck movements and respiratory activity. Two FBG sensors were encapsulated into a flexible matrix to have a robust system, and to improve the adherence of the sensing elements to the skin and the compliance with the neck movements. FBGs were chosen because of their lightweight, small size, high metrological performances, and immunity to electromagnetic interference [21,40,41,42,43,44,45,46]. A pilot study on five volunteers aimed at assessing the proposed wearable system in following FE and axial rotation (AR) of the neck, and f_R_. We focused on these two movements because prolonged head FE and twisted neck are the main vicious computer workers’ habits and on f_R_ because negatively influenced by job stress and wrong neck positions [16]. Therefore, an unobtrusive system providing quantitative information on the mentioned parameters may be helpful to prevent the effects of prolonged wrong postures and inadequate positions on computer workers. Future work will be focused on applying the wearable system on patients affected by MSDs to find out its potential to detect wrong postures and pathological conditions.

## 2. Description of the Wearable System

### 2.1. Flexible Sensors Based on FBG

**Figure 1:**
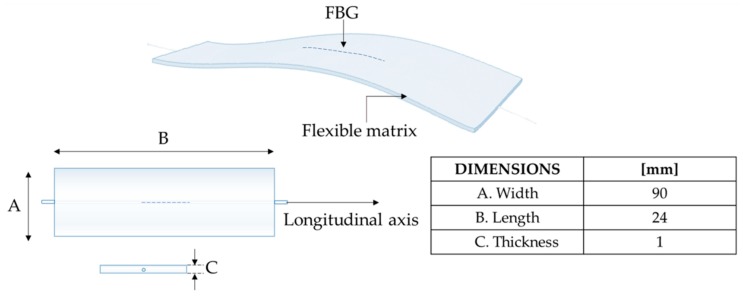
Design and dimensions of the fiber Bragg grating (FBG)-based flexible sensor.

The proposed wearable system consists of two flexible sensors based on FBG technology (i.e., FBG1 and FBG2 with Bragg wavelength λ_B_^FBG1^ of 1545 nm and λ_B_^FBG2^ of 1541 nm, grating length of 10 mm and reflectivity of 90%; AtGrating Technologies, Shenzen, China). Each flexible sensor encapsulates an FBG into a rectangular shaped matrix (approximately 90 mm × 24 mm × 1 mm) made of silicone rubber (Dragon Skin^TM^ 20; Smooth On, Inc., Macungie, PA, USA), as shown in Figure 1. The design, the manufacturing process, and the metrological properties of the flexible sensors are detailed in [42].

The working principle of the proposed system takes advantage of the FBG sensitivity to strain. An FBG is a diffraction grating, whose effect is to reflect a narrow part of the broadband incident spectrum. The peak of the narrowband spectral component, the λ*_B_* wavelength, relies on the effective refractive index of the fiber (*η_eff_*) and the period of the grating (Λ) as:

(1) $$\lambda_{B}=2\times \eta_{eff}\times \Lambda$$

Any external agent responsible for a longitudinal strain (*ε*) and/or a temperature variation (Δ*T*) may change both Λ and *η_eff_* resulting in a *λ_B_* shift (Δ*λ_B_*). Therefore, an FBG is intrinsically sensitive to both ε and *T* [47]:

(2) $$\frac{\Delta \lambda_{B}}{\lambda_{B}}=\left(1-p_{e}\right)\times \varepsilon +\left(\left(1-p_{e}\right)\times \alpha_{\Lambda }+\alpha_{n}\right)\times \Delta T$$

with *p_e_* the effective strain optic coefficient, α_Λ_ the fiber thermal expansion coefficient, and *α_n_* the fiber thermo-optic coefficient. Focusing on the application of interest, neck movements, and breathing activity mainly cause ε, and in turn Δ*λ_B_*, since temperature contribution can be assumed negligible.

### 2.2. Sensors Positioning and Measurement Parameters

Sensors positioning was carefully evaluated to ensure high sensor capability in detection and discrimination of different neck movements and breathing activity. A polyacrylate bandage (100% polyester, Curafix^®^ H, Lohmann & Rauscher, Padova, Italy) was used to allow a better adhesion and compliance to the skin. This bandage features adhesiveness, elasticity, and high breathability.

**Figure 2:**
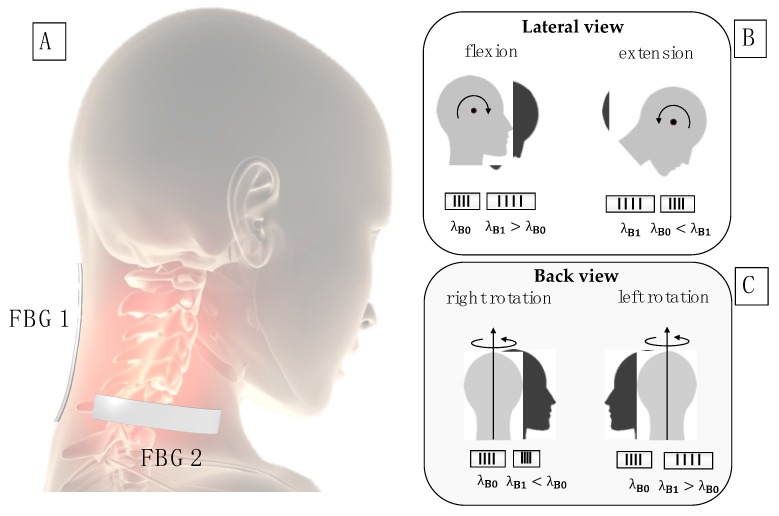
(**A**) Sensors’ positions; (**B**) schematic representation of the FBG1 response to flexion-extension (FE) movements; (**C**) schematic representation of the FBG2 response to axial rotation (AR) movements.

To detect FE movements, FBG1 was positioned in correspondence of the cervical spine segment C1–C7, along the longitudinal direction starting from C7. For the AR monitoring, FBG2 was positioned on the right side of the neck, horizontally with respect to FBG1 starting from the center of C6,C7 (Figure 2A).

The positions were chosen to optimize the strain distribution along the FBG longitudinal direction. The FE movements cause longitudinal strain on FBG1: its output increases during flexion from λ_B0_ to λ_B1_, while decreases during extension from λ_B1_ to λ_B0_ (Figure 2B). The AR movements are mostly detected by the FBG2: its output increases during left rotations, while a decrease is experienced during the right rotations (Figure 2C).

Breathing monitoring is allowed by the neck muscles activity and cervicothoracic junction movements that strain both the FBGs. This phenomenon results in Δ*λ_B_* pseudo-periodic oscillations, which allows estimating f_R_ [48].

## 3. System Assessment

### 3.1. Experimental Set-Up and Protocol

In this pilot study, a total of five healthy subjects (three males and two females) were enrolled. The subjects did not show any MSD or neck pain. Age and anthropometric measures (i.e., height, body mass, neck circumference) of each participant were collected before starting the experimental tests (Table 1).

**Figure 3:**
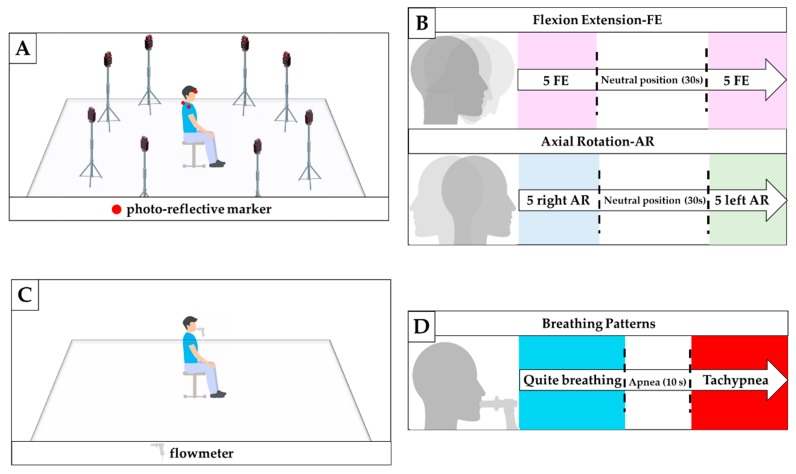
(**A**) Experimental set-up to assess the feasibility of the proposed system for monitoring neck movement; (**B**) experimental protocol for monitoring FE and AR movements; (**C**) Experimental set-up to assess the feasibility of the proposed system for respiratory frequency monitoring; (**D**) experimental protocol for f_R_ monitoring.

**Figure 4:**
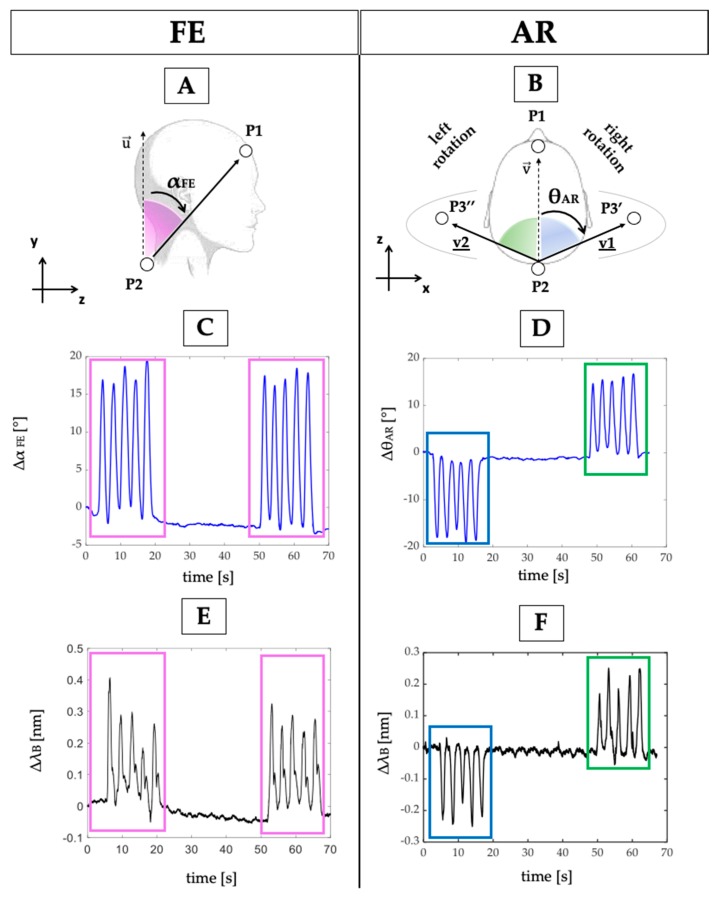
(**A**) The α_FE_ and (**B**) the θ_AR_ angles formed in the sagittal and transverse plane; (**C**) reference output changes over time during FE and (**D**) AR repetitions; (**E**) FBGs outputs changes over time during FE and (**F**) AR repetitions.

Participants were asked to sit on an armless and height-adjustable chair maintaining their feet on the floor, both hands on the knees, with hips and knees flexed at 90° (Figure 3A). A Motion Capture (MoCap) system (Smart-D, BTS Bioengineering Corp., Milan, Italy) was used as a gold standard to assess the capability of the multi-parametric wearable system to discriminate FE and AR movements. Four spherical, infrared photo-reflective markers (15.2 mm in diameter) were placed on each subject as shown in Figure 4A,B. In particular, the first marker is placed on the forehead (marker P1 in Figure 4A,B), the second marker on the C7 spinous process (marker P2 in Figure 4A,B), and the last two markers were placed in correspondence of the acromioclavicular joints (P3′ and P3″ in Figure 4B). 8 cameras collected the trajectories of the markers at a sampling rate of 60 Hz. An FBG interrogator (si255 based on HYPERION platform; Micron Optics Inc., Atlanta, GA, USA) was used to record the FBGs output, at a sampling rate of 1000 Hz.

After markers and FBGs positioning, the protocol was explained to each subject. Participants started with the head and neck in a neutral position and looking forward. Firstly, the participants were asked to perform FE and AR movements, simultaneously recorded by the wearable and MoCap systems. Each participant performed: (i) five FE repetitions, followed by 30 s in the neutral position, and then five FE repetition (Figure 3B); (ii) five AR repetitions to the right, followed by 30 s in the neutral position, and then by five AR repetition to the left (Figure 3B). An additional trial was executed to assess the ability of the multi-parametric wearable system to monitor breathing activity (Figure 3C). During this trial, a commercial flowmeter (SpiroQuant P, EnviteC, Alter Hozhafen, Wismar, Germany) connected to a differential pressure sensor (163PC01D75, Honeywell, Minneapolis, MN, USA) was used as a reference system. The output of the differential pressure sensor was collected through a DAQ (NI USB-6009, National Instrument, Rockville, MD, USA) and a custom Virtual Instrument developed in LabVIEW^®^ environment, at the sampling frequency of 250 Hz. Participants were asked to ventilate into a mouthpiece while performing two breathing patterns: ten breaths of self-controlled quite breathing and ten breaths of self-controlled tachypnea; a 10 s stage of apnea was performed between the two breathing patterns (Figure 3D).

### 3.2. Data Analysis

#### 3.2.1. Neck Movements

The number of FE and AR repetition was calculated from the raw data collected by the MoCap and the wearable systems.

The 3D coordinates of the markers recorded by the MoCap system were used to carry out the reference signals by following these steps: (i) the FE angle (α_FE_) was estimated in the sagittal plane (i.e., y–z) as the angle between the vectors $\vec{P2P1}$ and $\vec{u}$ (same direction of the *y*-axis), as shown in Figure 4A; (ii) the AR angle (θ_AR_) was estimated in the transverse plane (i.e., x–z,) as the angle between the vectors $\vec{P2P3^{\prime }}$ and $\vec{v}$ (the same trend may be obtained by considering the vectors $\vec{P2P3^{\prime \prime }}$ and $\vec{v}$), as shown in Figure 4B. The θ_AR_ decreases during the right rotation (clockwise) and increases during the left rotation (counterclockwise).

Regarding the wearable system, the analysis of the neck movements’ detection was performed as follows: (i) the changes of FBG1 output were used to evaluate FE movements since the chin lowered down toward the chest causes a longitudinal deformation of FBG1; (ii) the changes of FBG2 output were considered to evaluate AR movements, as the right and left rotations of the head around its vertical line (*y*-axis in Figure 4A) causes a longitudinal deformation of FBG2. 

Trends of signals collected by the MoCap system are shown in Figure 4C,D, and the ones collected by the wearable system are shown in Figure 4E,F.

**Figure 5:**
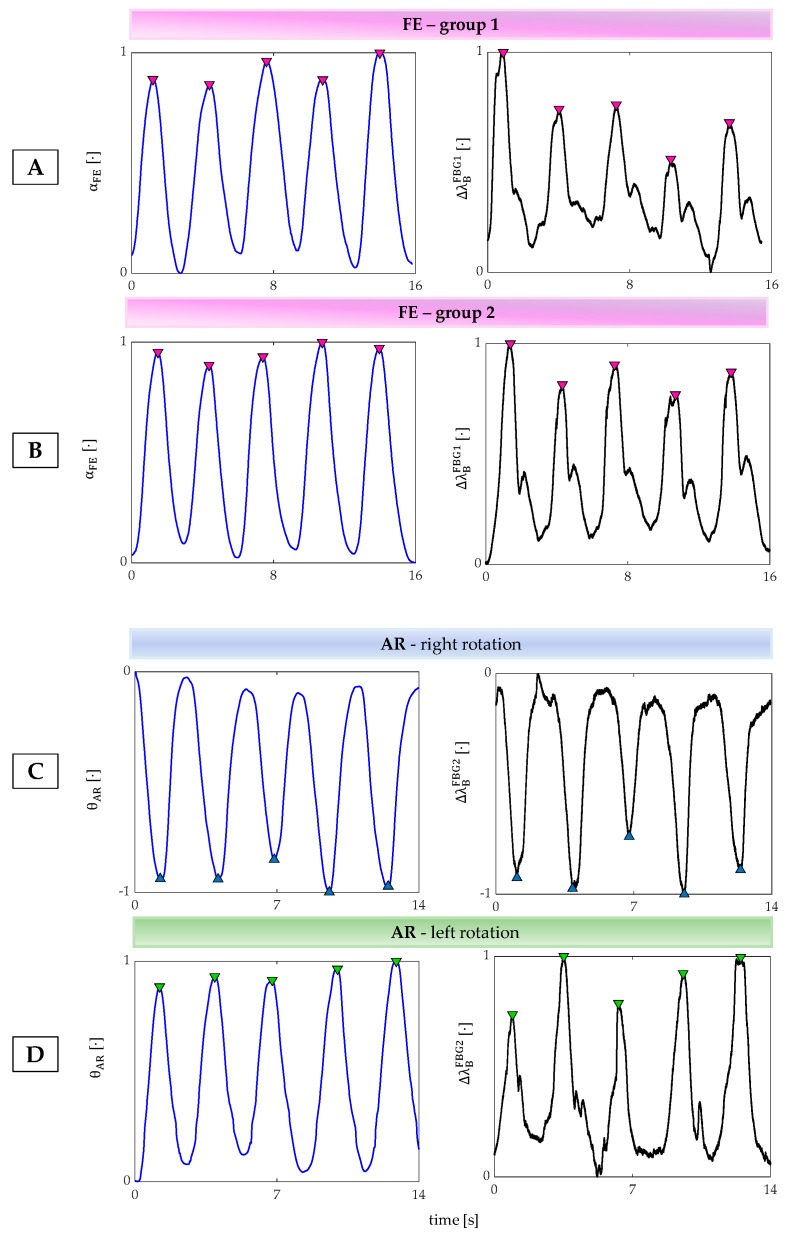
(**A**) The peak detection during the first group 5 FE, (**B**) the second group 5 FE right, (**C**) right AR repetitions, (**D**) left AR repetitions.

To assess the capability of the proposed system to detect neck movements on different planes, the collected data were processed by following two main steps:

1. the outputs of both the wearable and the MoCap systems were normalized in amplitude and plotted over time to evaluate trend similarity between signals;
2. the FE and AR repetitions were detected by using a custom peak detection algorithm in MATLAB environment. FE movements were detected by considering the maximum peaks of both MoCap and FBG1 signals: when $\alpha_{\mathrm{FE}}$ increases during the neck flexion (signal provided by the MoCap) FBG1 is strained with a consequent increase of *λ_B_* (Figure 5A,B). Right AR movements were detected by considering the minimum peaks of both MoCap and FBG2 signals: when θ*_AR_* decreases during the right AR (signal provided by the MoCap) FBG2 is compressed with a consequent decrement of *λ_B_* (Figure 5C). These data were collected during the first 5 AR repetitions; left AR movements were detected by considering the maximum peaks of both MoCap and FBG2 signals because when θ*_AR_* increases during the left AR (signal provided by the MoCap) FBG2 is strained with a consequent increment of *λ_B_* (Figure 5B). These data were collected during the last 5 AR repetitions.

#### 3.2.2. Breathing Activity

**Figure 6:**
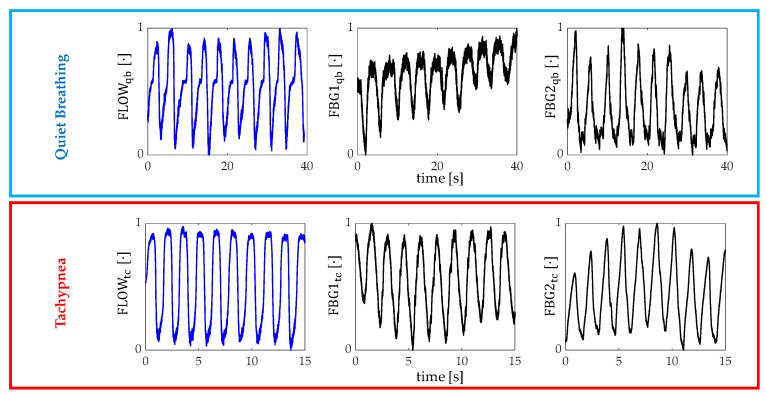
Signals collected by the flowmeter (blue trend) and by the FBGs (black trend) during both quiet breathing (light blue box) and tachypnea (red box).

**Figure 7:**
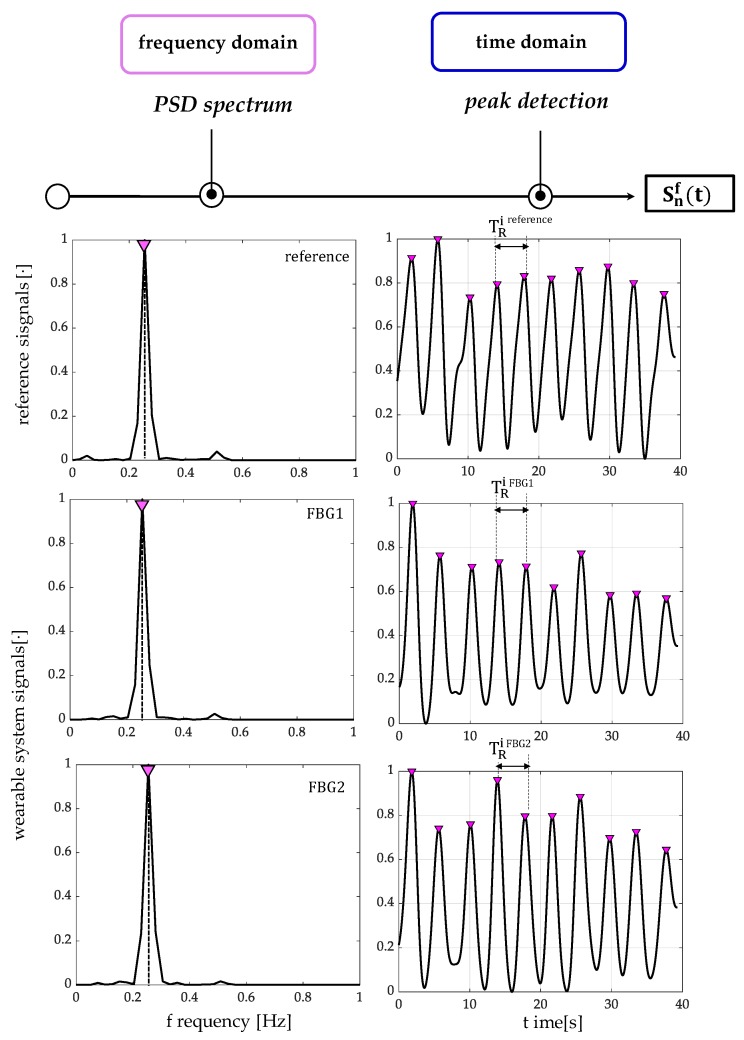
An example of signals processing performed for the f_R_ estimation from data recorded by the flowmeter and the wearable systems, during quiet breathing. The power spectral density (PSD) spectra over frequency [Hz] and the peak detection over time [s] are shown for both the reference system and the proposed wearable system based on two flexible sensors (FBG1 and FBG2). The S^f^_n_ (t) signals are filtered and normalized.

The assessment of the proposed wearable system for the *f_R_* monitoring was performed by using the flowmeter as a reference instrument and following six main steps:

1. The outputs of the wearable system and the flowmeter were normalized in amplitude and split into quiet breathing-related signals and tachypnea-related ones (i.e., FBG1_qb_, FBG2_qb_, FLOW_qb,_ FBG1_tc_, FBG2_tc_, and FLOW_tc_), as shown in Figure 6;
2. The signal of both FBG1_qb_ and FBG1_tc_ were inverted since the FBG1 was compressed during the inspiration (when the volume of lungs increases) and tensioned during the expiration (when the volume of lungs decreases). This step was not implemented on the FBG2 output since its trend in time matches that of the reference system;
3. a third-order Butterworth low pass filter was applied on signals collected during quiet breathing (cut-off frequency, fc, of 0.5 Hz) and during tachypnea (fc of 3 Hz);
4. spectral analysis in terms of power spectral density (PSD) was performed on the filtered signals and the maximum frequency (f_0_) of both the reference and the wearable systems signals were evaluated (Figure 7);
5. peak detection was performed by using *findpeaks* in MATLAB environment: the input parameter related to minimum peaks distance was set starting from the value of f_0_ (Figure 7);
6. the respiratory periods of each breath (i.e., T_R_^i^) was computed as the time elapsed between two consecutive maximum peaks of the signal provided by FBG1, FBG2, and the flowmeter, see Figure 7. The f_R_^i^ values during both quiet breathing and tachypnea were estimated as 60/T_R_^i^ and expressed as breaths per minute (bpm).

The assessment of the wearable system in the estimation of *f_R_* during both quiet breathing and tachypnea was performed using three parameters:

1. in terms of percentage error ($e_{p}$) as in:
  (3) $$e_{p}\;\left[\%\right]=\frac{{\bar{f_{R}}}^{FBG}-{\bar{f_{R}}}^{FLOW}}{{\bar{f_{R}}}^{FLOW}}\times 100$$
  where $\bar{f_{R}}$ is the mean value of *f_R_*;
2. in terms of absolute percentage errors for a breath-by-breath analysis:
  (4) $$\left|e_{p}^{i}\right|\left[\%\right]=\frac{\left|f_{R}^{i}{}^{FBG}-f_{R}^{i}{}^{FLOW}\right|}{f_{R}^{i}{}^{FLOW}}\times 100$$
  where $f_{R}^{i}{}^{FBG}$ and $f_{R}^{i}{}^{FLOW}$ are the values of the *i*th *f_R_* estimated either by FBG1 or FBG2 and by the flowmeter;
3. by calculating the mean value of the breath-by-breath absolute percentage errors (i.e., MAPE), for each volunteer as in:
  (5) $$\left|e_{p}^{i}\right|\;\left[\%\right]=\frac{1}{n}\cdot \sum^{\;}\frac{\left|f_{R}{}^{FBG}-f_{R}{}^{FLOW}\right|}{f_{R}{}^{FLOW}}\cdot 100$$

### 3.3. Results

#### 3.3.1. Detection of Neck Movements

**Figure 8:**
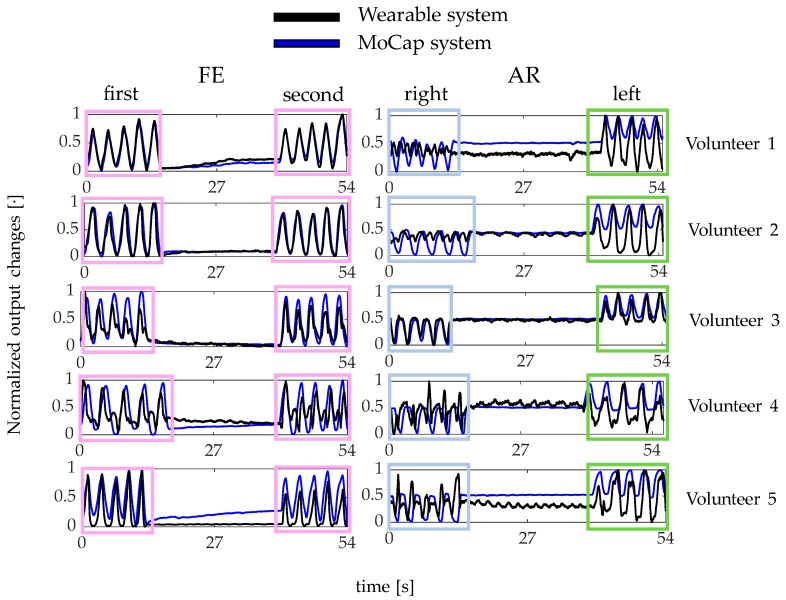
The output changes of both the wearable (black line) and the Motion Capture (MoCap) system (blue line) collected during FE and AR repetitions.

Results showed that the proposed wearable system was able to follow both FE and AR movements and detect the repetitions, as shown in Figure 8. 

In particular, the wearable system showed good performance in detecting FE and left AR repetitions. Indeed, the Δ*λ_B_*^FBG1^ and Δ*λ_B_*^FBG2^ patterns matched the MoCap ones (pink and light blue boxes in Figure 8). On the contrary, Δ*λ_B_*^FBG2^ pattern during the right AR movements did not always match the reference signal (green box in Figure 8).

#### 3.3.2. Breathing Activity: Respiratory Frequency Estimation

**Figure 9:**
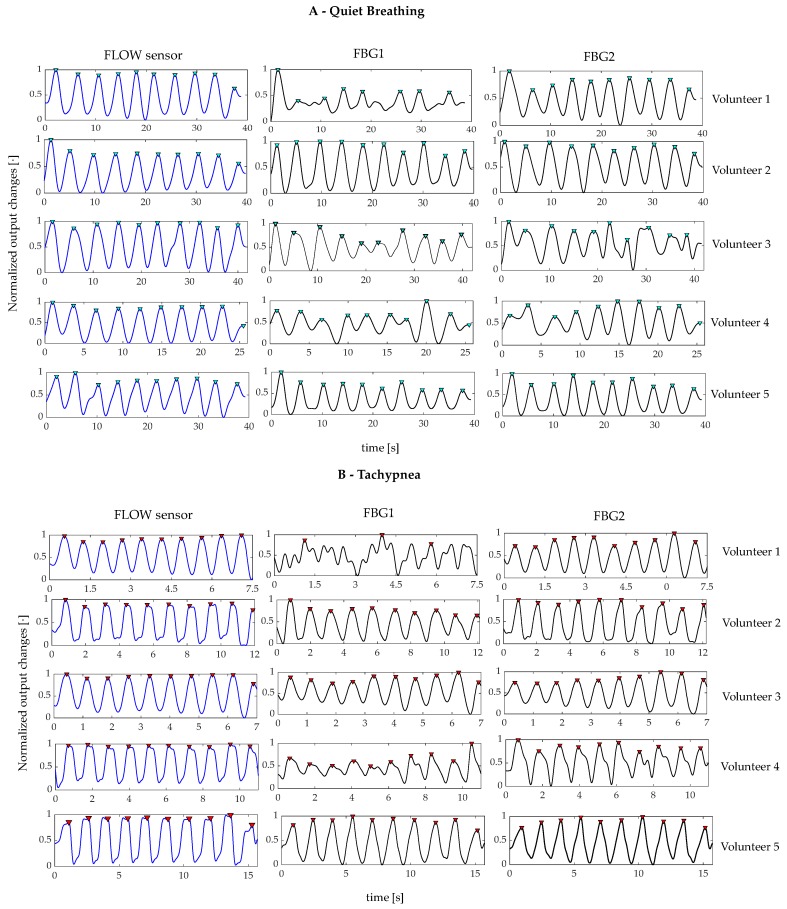
(**A**) signals collected by the flowmeter (blue line) and the FBGs (black lines) for each volunteer during quiet breathing and (**B**) during tachypnea. All the signals are synchronized, filtered, and normalized. The detected peaks are highlighted by using red markers.

All signals involved in the peak detection of the breathing analysis are shown in Figure 9. The peak detection allowed estimating *f_R_* in all volunteers but one (for FBG1 output changes of Volunteer 1 during both quiet breathing and tachypnea).

The e_p_, the MAPE and the |e_p_| values are listed in Table 2 and Table 3. The e^FBG1^ are always ≤1.53% and ≤0.71% whereas the e^FBG2^ ≤ 6.09% and ≤1.90%, during quiet breathing and tachypnea, respectively. The MAPE^FBG1^ errors are always ≤12.87% and ≤5.86%, and MAPE^FBG2^ always ≤15.36% and ≤4.90%, during quiet breathing and tachypnea, respectively. Data from FBG1 for Volunteer 1 were discarded.

## 4. Discussion

In this pilot study, a multi-parametric wearable system was used to detect both neck movements (i.e., FE, and AR) and f_R_ in computer workers. The system consists of two custom flexible sensors based on FBG technology (i.e., FBG1 and FBG2). Each FBG was encapsulated into a silicone matrix which improves the FBGs robustness, adherence to the skin, and compliance with the neck movements. Moreover, the flexibility provided by the encapsulation enhances the FBGs usability making them more competitive than other sensors in some medical applications [49].

This is the first study reporting on a wearable system able to monitor the abovementioned parameters, which significantly expands our explorative study on a single FBG-based wearable system [39]. Indeed, the presence of 2 FBGs allowed the new system to monitor both neck movements and f_R_. In addition, we performed a quantitative assessment of system performances on five volunteers_,_ by using reference systems during each trial. 

Regarding the neck movements’ detection, the proposed wearable system showed good performance in following both FE and left AR movements and detect the repetitions, while some limitations resulted in the right AR detection. These findings could be explained considering different working conditions of FBG2 during AR repetitions: the grating is tensioned during left AR and compressed during right AR. Therefore, the FBG2 compression during right AR causes a partially adherence of this sensor to the neck surface. As a consequence, the asymmetric sensor arrangements can cause small distortions of the reflection spectrum [50]. In the literature, the neck movements’ detection was mainly performed by using wearable systems based on electric sensors (e.g., inertial sensors [36], accelerometers [37], and piezoresistive sensors [38]). Two inertial sensors were proposed to evaluate FE, AR, and lateral bending (LB) of patients treated with cervical arthrodesis [36]. Sensors were placed on the forehead and on the sternum, respectively, and an optoelectronic system was used as a reference instrument. With respect to our system, such wearable inertial sensors required a pre-calibration to align the sensor axis with the segment anatomical frame. Moreover, the measurement units were not located on the neck but on single points of other anatomical segments. A 3-axis accelerometer was placed on the forehead to monitor cervical postures [37]. Only FE movements were monitored but no reference instrument was used to assess such capability. The flexible encapsulation of our sensors allows for a multi-point positioning and better compliance with the neck anatomy with respect to these solutions based on accelerometers or inertial sensors. A wearable system based on six piezoresistive sensors was proposed to monitor FE, AR, and LB movements [38]. As our system, the sensing elements detected neck movements from the induced strain, being in direct contact with the skin. Each movement was monitored by using a couple of sensors on the opposite sides of the neck whereas we used only one FBG for FE and one FBG for AR. Our choice was motivated by the desire to enhance system wearability and comfortability. Results in the literature [38] suggested that placing sensors diametrically opposite on the neck can allow monitoring both right and left AR movements despite the higher amount of wires.

Further developments of our system can address the wiring issue thanks to the multiplexing FBGs capability. In all these studies, the monitoring of breathing activity was not taken into account, although evidences suggested a relationship between neck pain and respiratory disfunction [16,17]. Moreover, f_R_ is sensitive to cognitive load and emotions with important implications for workers exposed to highly demanding tasks [18,19,20].

The high sensitivity of the custom made flexible FBGs allows our system the monitoring of f_R_ from the neck. Our findings suggest a good accuracy in f_R_ monitoring in terms of mean and breath-by-breath values in all trials but one (i.e., Volunteer1). In this trial, FBG1 failed during both quiet breathing and tachypnea, presumably because of a non-well adherence of the sensing element to the skin due to a more prominent C1–C7 cervical segment and skin surface properties. For all the other volunteers, both FBG1 and FBG2 were able to detect f_R_ values. They showed comparable results in terms of mean and breath-by-breath values during both quiet breathing and tachypnea (i.e., e_p_ ≤ 6.09% vs. ≤1.90%, |e_p_| ≤ 29.36% vs. ≤18.86%%, and MAPE ≤15.36% vs. ≤5.86% during quiet breathing and tachypnea, respectively). Similar results between f_R_ values estimated by FBG1 and FBG2 are confirmed by considering the agreement with respect to the reference instrument (e.g., MAPE^FBG1^ ≤12.87% vs. MAPE^FBG2^ ≤ 15.36% during quiet breathing, and MAPE^FBG1^ ≤ 5.86% vs. MAPE^FBG2^ ≤ 4.90%, during tachypnea).

In the literature, the majority of wearables for f_R_ monitoring used strain sensors located at the chest surface [42,51,52,53] Only a few studies investigated the possibility of monitoring respiratory activity by using acoustic sensors in contact with the neck [54,55]. An acoustic sensor was attached to the anterior lateral base of the neck to measure the sounds coming from the flow of air in the trachea [54]. Each of the five volunteers enrolled in the study was instructed to breathe slowly, passing to quiet breathing and ending with tachypnea. A commercial acoustic transducer (RRa^TM^ rev C, Masimo Corp, Irvine, CA, USA) was applied to the patient’s throat and connected to a monitor (Rad-87 Pulse CO-Oximeter, Masimo Corp.) [55]. These acoustic systems are usually employed in sound-controlled environments and need the rejection of noised signals related to heartbeat, muscle activations, and swallowing [56].

## 5. Conclusions

In conclusion, we reported an FBG-based multi-parametric wearable system which can be considered a first attempt to monitor both neck FE and AR movements since highly affected by the harmful postural habits of computer, and f_R_ since the evident correlation between wrong neck postures with respiratory dysfunctions. The present study is intended to be a pilot study in which five healthy volunteers were enrolled, both males and females and the capability of neck movements detection and fR monitoring was assessed. The strength of the proposed multi-parametric wearable system relies on the capability to provide multiple measures that could have a great impact in the occupational health and safety interventions. Further tests will be devoted to increasing the sample size and enrolling patients suffering from neck pain to figure out if the system is able to discriminate pathological conditions from healthy ones. More FBGs will be added to improve the system capability of monitoring neck movements (including also LB) and study the influence of different anthropometric characteristics on the FBGs output. Finally, the capability of the system to estimate neck range of motion during FE, AR and LB movements will be investigated.

## Figures and Tables

**Table 1:** Participants characteristics.

	Age (Years)	Height (cm)	Body Mass (kg)	Neck Circumference (cm)
Volunteer 1	23	183	125	46
Volunteer 2	28	171	61	32
Volunteer 3	27	178	85	38
Volunteer 4	31	163	60	38
Volunteer 5	39	171	71	43

**Table 2:** Mean f_R_ values and percentage errors (e_P_).

**Quiet Breathing**			
**Volunteer**	$\bar{f_{R}}\;$ **^FBG1^ [bpm]**	$\bar{f_{R}}$ **^FLOW^ [bpm]**	${\bar{e}}_{p}^{FBG1}$ **[%]**
1	-	15.37	-
2	14.47	14.63	−1.09
3	14.36	14.14	1.53
4	22.36	22.09	1.22
5	15.15	15.22	−0.45
	$\bar{f_{R}}$ **^FBG2^ [bpm]**	$\bar{f_{R}}$ **^FLOW^ [bpm]**	${\bar{e}}_{p}^{FBG2}$ **[%]**
1	15.30	15.37	−0.48
2	14.65	14.63	0.15
3	15.00	14.14	6.09
4	22.40	22.09	1.40
5	15.10	15.22	−0.79
**Tachypnea**			
**Volunteer**	$\bar{f_{R}}$ **^FBG1^ [bpm]**	$\bar{f_{R}}$ **^FLOW^ [bpm]**	${\bar{e}}_{p}^{FBG1}$ **[%]**
1	-	83.62	-
2	48.65	48.80	−0.32
3	83.28	83.87	−0.71
4	54.53	54.58	−0.09
5	37.88	38.09	−0.56
	$\bar{f_{R}}$ **^FBG2^ [bpm]**	$\bar{f_{R}}$ **^FLOW^ [bpm]**	${\bar{e}}_{p}^{FBG2}$ **[%]**
1	82.02	83.62	−1.90
2	48.46	48.80	−0.72
3	82.98	83.87	−1.05
4	54.99	54.58	0.75
5	38.01	38.09	−0.19

**Table 3:** Breath-by-breath absolute percentage errors, |e_p_|, and mean absolute percentage error (MAPE) values.

	**Quiet Breathing**									
**Volunteer**	**|e_p_^FBG1^| [%]**									**MAPE^FBG1^ [%]**
1	-	-	-	-	-	-	-	-	-	-
2	10.40	0.34	0.28	0.19	0.58	1.91	2.24	2.93	3.22	2.45
3	16.66	10.88	2.28	4.97	10.26	7.75	13.7	5.62	8.17	8.92
4	12.32	7.11	14.04	13.51	10.17	28.65	1.24	15.56	13.21	12.87
5	0.21	8.24	4.48	5.92	0.91	3.56	6.58	4.71	1.04	1.47
	**|e_p_^FBG2^| [%]**									**MAPE^FBG2^ [%]**
1	5.28	3.76	0.20	2.18	4.75	4.19	1.33	4.92	4.33	3.44
2	2.69	0.86	2.06	1.14	0.97	2.09	0.19	6.76	4.42	2.36
3	29.36	12.12	4.05	3.63	17.35	27.82	8.21	15.75	19.93	15.36
4	15.34	16.03	4.22	0.85	7.36	3.46	3.33	0.16	1.35	5.79
5	2.53	1.52	0.95	1.95	~0	0.41	2.31	2.89	1.23	1.53
	**Tachypnea**									
**Volunteer**	**|e_p_^FBG1^| [%]**									**MAPE^FBG1^ [%]**
1	-	-	-	-	-	-	-	-	-	-
2	3.74	0.65	3.11	3.34	7.56	6.21	4.64	11.54	3.75	4.95
3	1.69	2.70	1.71	0.55	3.72	4.09	~0	5.82	1.16	2.38
4	2.95	9.77	4.48	18.86	0.37	1.46	1.44	5.35	8.10	5.86
5	0.52	1.06	0.77	1.84	0.50	0.73	0.24	1.31	2.11	1.01
	**|e_p_^FBG2^| [%]**									**MAPE^FBG2^ [%]**
1	6.95	2.26	0.56	2.72	2.63	3.68	1.63	2.81	5.13	3.15
2	4.07	0.97	3.67	2.52	3.34	0.96	0.97	6.69	3.45	2.96
3	8.85	7.14	0.55	~0	1.12	1.14	0.55	1.11	7.41	3.10
4	6.41	2.47	9.92	5.24	5.43	2.11	6.23	6.29	~0	4.90
5	0.52	1.33	0.26	1.84	0.49	~0	1.72	1.02	0.72	0.88

## References

[1] Ailneni R.C., Syamala K.R., Kim I.-S., Hwang J. (2019). Influence of the wearable posture correction sensor on head and neck posture: Sitting and standing workstations. Work.

[2] Barbieri D.F., Srinivasan D., Mathiassen S.E., Oliveira A.B. (2019). Variation in upper extremity, neck and trunk postures when performing computer work at a sit-stand station. Appl. Ergon..

[3] Bevan S. (2015). Economic impact of musculoskeletal disorders (MSDs) on work in Europe. Best Pract. Res. Clin. Rheumatol..

[4] Näf M.B., Koopman A.S., Baltrusch S., Rodriguez-Guerrero C., Vanderborght B., Lefeber D. (2018). Passive back support exoskeleton improves range of motion using flexible beams. Front. Robot. AI.

[5] Jun D., Zoe M., Johnston V., O’Leary S. (2017). Physical risk factors for developing non-specific neck pain in office workers: A systematic review and meta-analysis. Int. Arch. Occup. Environ. Health.

[6] Ekinci Y., Uysal S.A., Kabak V.Y., Duger T. (2019). Does ergonomics training have an effect on body posture during computer usage?. J. Back Musculoskelet. Rehabil..

[7] Kennedy C.A., Amick B.C., Dennerlein J.T., Brewer S., Catli S., Williams R., Serra C., Gerr F., Irvin E., Mahood Q. (2010). Systematic review of the role of occupational health and safety interventions in the prevention of upper extremity musculoskeletal symptoms, signs, disorders, injuries, claims and lost time. J. Occup. Rehabil..

[8] Cunha B., Tadi P., Bragg B.N. (2019). Congenital Torticollis. StatPearls.

[9] Jaturongkhasumrit K., Mekhora K., Somprasong S. (2019). Immediate Effect of Stress-induced Computer Typing on EMG Activity of Accessory Breathing Muscles and Respiratory Rate. J. Public Health.

[10] Shikdar A.A., Al-Kindi M.A. (2007). Office ergonomics: Deficiencies in computer workstation design. Int. J. Occup. Saf. Ergon..

[11] Elwardany S.H., El-Sayed W.H., Ali M.F. (2015). Reliability of Kinovea computer program in measuring cervical range of motion in sagittal plane. OALib.

[12] Kang B.-R., Her J.-G., Lee J.-S., Ko T.-S., You Y.-Y. (2019). Effects of the Computer Desk Level on the Musculoskeletal Discomfort of Neck and Upper Extremities and EMG Activities in Patients with Spinal Cord Injuries. Occup. Ther. Int..

[13] Intolo P., Shalokhon B., Wongwech G., Wisiasut P., Nanthavanij S., Baxter D.G. (2019). Analysis of neck and shoulder postures, and muscle activities relative to perceived pain during laptop computer use at a low-height table, sofa and bed. Work.

[14] Mekhora K., Liston C.B., Nanthavanij S., Cole J.H. (2000). The effect of ergonomic intervention on discomfort in computer users with tension neck syndrome. Int. J. Ind. Ergon..

[15] Saravanan K., Kumar I.P. (2019). Effects of Ergonomic Training and Active Exercises for Non–Specific Work Related Upper Extremity Musculoskeletal Disorders in Women Working in Video Display Units. Asian J. Orthop. Res..

[16] Kahlaee A.H., Ghamkhar L., Arab A.M. (2017). The Association between Neck Pain and Pulmonary Function: A Systematic Review. Am. J. Phys. Med. Rehabil..

[17] Zafar H., Albarrati A., Alghadir A.H., Iqbal Z.A. (2018). Effect of different head-neck postures on the respiratory function in healthy males. Biomed Res. Int..

[18] Grassmann M., Vlemincx E., von Leupoldt A., Mittelstädt J.M., Van den Bergh O. (2016). Respiratory changes in response to cognitive load: A systematic review. Neural Plast..

[19] Grassmann M., Vlemincx E., von Leupoldt A., Van den Bergh O. (2016). The role of respiratory measures to assess mental load in pilot selection. Ergonomics.

[20] Tipton M., Harper A., Paton J.F.R., Costello J.T. (2017). The human ventilatory response to stress: Rate or depth?. J. Physiol..

[21] Massaroni C., Nicolò A., Lo Presti D., Sacchetti M., Silvestri S., Schena E. (2019). Contact-Based Methods for Measuring Respiratory Rate. Sensors.

[22] Massaroni C., Lopes D.S., Lo Presti D., Schena E., Silvestri S. (2018). Contactless monitoring of breathing patterns and respiratory rate at the pit of the neck: A single camera approach. J. Sensors.

[23] Malasinghe L.P., Ramzan N., Dahal K. (2019). Remote patient monitoring: A comprehensive study. J. Ambient Intell. Humaniz. Comput..

[24] Massaroni C., Venanzi C., Silvatti A.P., Lo Presti D., Saccomandi P., Formica D., Giurazza F., Caponero M.A., Schena E. (2018). Smart textile for respiratory monitoring and thoraco-abdominal motion pattern evaluation. J. Biophotonics.

[25] Khan Y., Ostfeld A.E., Lochner C.M., Pierre A., Arias A.C. (2016). Monitoring of vital signs with flexible and wearable medical devices. Adv. Mater..

[26] Carnevale A., Longo U.G., Schena E., Massaroni C., Presti D.L., Berton A., Candela V., Denaro V. (2019). Wearable systems for shoulder kinematics assessment: A systematic review. BMC Musculoskelet. Disord..

[27] Gong T., Zhang H., Huang W., Mao L., Ke Y., Gao M., Yu B. (2018). Highly responsive flexible strain sensor using polystyrene nanoparticle doped reduced graphene oxide for human health monitoring. Carbon N. Y..

[28] Xu H., Lu Y.F., Xiang J.X., Zhang M.K., Zhao Y.J., Xie Z.Y., Gu Z.Z. (2018). A multifunctional wearable sensor based on a graphene/inverse opal cellulose film for simultaneous, in situ monitoring of human motion and sweat. Nanoscale.

[29] Papi E., Koh W.S., McGregor A.H. (2017). Wearable technology for spine movement assessment: A systematic review. J. Biomech..

[30] Voinea G.-D., Butnariu S., Mogan G. (2017). Measurement and geometric modelling of human spine posture for medical rehabilitation purposes using a wearable monitoring system based on inertial sensors. Sensors.

[31] Fathi A., Curran K. (2017). Detection of spine curvature using wireless sensors. J. King Saud Univ. Sci..

[32] O’Sullivan K., O’Sullivan L., Campbell A., O’Sullivan P., Dankaerts W. (2012). Towards monitoring lumbo-pelvic posture in real-life situations: Concurrent validity of a novel posture monitor and a traditional laboratory-based motion analysis system. Man. Ther..

[33] Williams J.M., Haq I., Lee R.Y. (2010). Dynamic measurement of lumbar curvature using fibre-optic sensors. Med. Eng. Phys..

[34] Dunne L.E., Walsh P., Hermann S., Smyth B., Caulfield B. (2008). Wearable monitoring of seated spinal posture. IEEE Trans. Biomed. Circuits Syst..

[35] Dunne L., Walsh P., Smyth B., Caulfield B. (2007). A system for wearable monitoring of seated posture in computer users. 4th International Workshop on Wearable and Implantable Body Sensor Networks (BSN 2007).

[36] Duc C., Salvia P., Lubansu A., Feipel V., Aminian K. (2014). A wearable inertial system to assess the cervical spine mobility: Comparison with an optoelectronic-based motion capture evaluation. Med. Eng. Phys..

[37] Wang Y., Zhou H., Yang Z., Samuel O.W., Liu W., Cao Y., Li G. An intelligent wearable device for human’s cervical vertebra posture monitoring. Proceedings of the 2018 40th Annual International Conference of the IEEE Engineering in Medicine and Biology Society (EMBC).

[38] Maselli M., Mussi E., Cecchi F., Manti M., Tropea P., Laschi C. (2018). A wearable sensing device for monitoring single planes neck movements: Assessment of its performance. IEEE Sens. J..

[39] Presti D.L., Massaroni C., Di Tocco J., Schena E., Carnevale A., Longo U.G., D’Abbraccio J., Massari L., Oddo C.M., Caponero M.A. Single-plane neck movements and respiratory frequency monitoring: A smart system for computer workers. Proceedings of the 2019 II Workshop on Metrology for Industry 4.0 and IoT (MetroInd4. 0&IoT).

[40] Dinia L., Mangini F., Muzi M., Frezza F. (2018). FBG multifunctional pH sensor-monitoring the pH rain in cultural heritage. Acta IMEKO.

[41] Tosi D., Poeggel S., Iordachita I., Schena E. (2018). Fiber Optic Sensors for Biomedical Applications. Opto-Mechanical Fiber Optic Sensors: Research, Technology, and Applications in Mechanical Sensing.

[42] Presti D.L., Massaroni C., D’Abbraccio J., Massari L., Caponero M., Longo U.G., Formica D., Oddo C., Schena E. (2019). Wearable system based on flexible FBG for respiratory and cardiac monitoring. IEEE Sens. J..

[43] Roriz P., Lobo Ribeiro A.B. (2018). Fiber Optical Sensors in Biomechanics. Opto-Mechanical Fiber Optic Sensors: Research, Technology, and Applications in Mechanical Sensing.

[44] Servati A., Zou L., Jane Wang Z., Ko F., Servati P. (2017). Novel flexible wearable sensor materials and signal processing for vital sign and human activity monitoring. Sensors.

[45] Lo Presti D., Massaroni C., Formica D., Giurazza F., Schena E., Saccomandi P., Caponero M.A., Muto M. Respiratory and cardiac rates monitoring during MR examination by a sensorized smart textile. Proceedings of the I2MTC 2017—2017 IEEE International Instrumentation and Measurement Technology Conference.

[46] Massaroni C., Saccomandi P., Formica D., Lo Presti D., Caponero M.A., Di Tomaso G., Giurazza F., Muto M., Schena E. (2016). Design and Feasibility Assessment of a Magnetic Resonance-Compatible Smart Textile Based on Fiber Bragg Grating Sensors for Respiratory Monitoring. IEEE Sens. J..

[47] Erdogan T. (1997). Fiber grating spectra. J. Light. Technol..

[48] Kang J.-I., Jeong D.-K., Choi H. (2018). Correlation between pulmonary functions and respiratory muscle activity in patients with forward head posture. J. Phys. Ther. Sci..

[49] Taffoni F., Formica D., Saccomandi P., Di Pino G., Schena E. (2013). Optical fiber-based MR-compatible sensors for medical applications: An overview. Sensors.

[50] Takahashi S., Hao J.Z., Lee Y.W.A., Cai Z., Do T.T., Ng B.Y.R. (2005). Effect of bending methods on FBG lateral force sensor. Electron. Lett..

[51] Dziuda Ł., Skibniewski F.W., Krej M., Baran P.M. (2013). Fiber Bragg grating-based sensor for monitoring respiration and heart activity during magnetic resonance imaging examinations. J. Biomed. Opt..

[52] Massaroni C., Di Tocco J., Presti D.L., Longo U.G., Miccinilli S., Sterzi S., Formica D., Saccomandi P., Schena E. (2019). Smart textile based on piezoresistive sensing elements for respiratory monitoring. IEEE Sens. J..

[53] Huang C.T., Shen C.L., Tang C.F., Chang S.H. (2008). A wearable yarn-based piezo-resistive sensor. Sens. Actuators Phys..

[54] Sierra G., Telfort V., Popov B., Pelletier M., Despault P., Agarwal R., Lanzo V. Comparison of respiratory rate estimation based on tracheal sounds versus a capnograph. Proceedings of the 2005 Annual International Conference of the IEEE Engineering in Medicine and Biology.

[55] Mimoz O., Benard T., Gaucher A., Frasca D., Debaene B. (2012). Accuracy of respiratory rate monitoring using a non-invasive acoustic method after general anaesthesia. Br. J. Anaesth..

[56] Corbishley P., Rodríguez-Villegas E. (2008). Breathing detection: Towards a miniaturized, wearable, battery-operated monitoring system. IEEE Trans. Biomed. Eng..

